# Neurobrucellosis in Stranded Dolphins, Costa Rica

**DOI:** 10.3201/eid1409.071056

**Published:** 2008-09

**Authors:** Gabriela Hernández-Mora, Rocío González-Barrientos, Juan-Alberto Morales, Esteban Chaves-Olarte, Caterina Guzmán-Verri, Elías Baquero-Calvo, María-Jesús De-Miguel, Clara-María Marín, José-María Blasco, Edgardo Moreno

**Affiliations:** Universidad Nacional, Heredia, Costa Rica (G. Hernández-Mora, R. González-Barrientos, J.-A. Morales, E. Chaves-Olarte, C. Guzmán-Verri, E. Baquero-Calvo, E. Moreno); Universidad de Costa Rica, San José, Costa Rica (E. Chaves-Olarte); Sanidad Animal, Gobiemo de Aragón, Aragón, Spain (M.-J. De-Miguel, C.-M. Marín, J.-M. Blasco)

**Keywords:** Brucella, Brucella ceti, brucellosis, dolphins, meningoencephalitis, neurobrucellosis, Stenella coeruleoalba, Costa Rica, zoonosis, dispatch

## Abstract

Ten striped dolphins, *Stenella coeruleoalba,* stranded along the Costa Rican Pacific coast, had meningoencephalitis and antibodies against *Brucella* spp. *Brucella ceti* was isolated from cerebrospinal fluid of 6 dolphins and 1 fetus. *S. coeruleoalba* constitutes a highly susceptible host and a potential reservoir for *B. ceti* transmission.

Brucellosis is a zoonotic disease of terrestrial and marine mammals. During the past 3 decades, contacts between cetaceans and humans have increased worldwide (*1*), augmenting the risk for transmission of pathogenic *Brucella* spp. from these animals to people (*2*). Indeed, *Brucella* marine strains are capable of infecting humans and livestock (*3*,*4*).

## The Study

From August 2004 through April 2007, 10 live striped dolphins, *Stenella coeruleoalba* (3 female adults, 2 female juveniles, 1 female calf, 4 juvenile males), were found stranded in populated areas at the Pacific shoreline of the Puntarenas Province of Costa Rica. All animals had swimming problems compatible with neurologic disorders and died within 48 hours of being found. Corpses were kept on ice and transported to the Pathology Unit, Veterinary School, National University, Costa Rica, for sampling; necropsy; and histopathologic, immunohistochemical, and serologic studies. With exception of 1 dwarf sperm whale, *Kogia sima*, these 10 dolphins were the only cetaceans we were able to examine during this 32-month period.

Because marine *Brucella* spp. have been reported to cause intracerebral infections (*3*), we decided to perform immunohistochemical and serologic tests. For these tests, rabbit immunoglobulin (Ig) G anti–*B. abortus* lipopolysaccharide (LPS) was produced and isolated as described elsewhere (*5*). Antibodies against dolphin *Steno bredanensis* IgG were produced in rabbits, purified according to described protocols (*6*). Both rabbit antibodies were linked to fluorescein isothiocyanate and peroxidase and were assayed by using immunofluorescent and Western blot techniques, respectively (*5*,*7*). Rose Bengal agglutination test, immunofluorescent assays, and competitive ELISA were designed and used as described (*8*,*9*).

Blood was collected from the live dolphins in situ, serum was obtained, and physical and chemical examinations were performed, followed after death by necropsies and gross pathologic and histopathologic studies. Tissues were fixed in formalin, embedded in paraffin wax, sectioned, and stained with hematoxylin and eosin (*10*). Organs and tissues of 5 adult females and 1 juvenile male were analyzed for bacteria (*11*); however, no samples for bacteriologic studies were available from the other dolphins that were stranded before July 2006. Identification of the bacterial isolates was performed according to standard protocols (*11*,*12*). Fresh tissue impressions or pellets from supernatants of macerated tissues were fixed with cold 3% paraformaldehyde for 15 min on ice and subjected to immunofluorescence for detection of *Brucella* spp. (*9*). Genotyping of *Brucella* isolates was performed by PCR, using 5′-GGCTGATCTCGCAAAGAT-3′ and 5′-CCAGGTCCTTGGCTTCCTTGAG-3′ primers (Invitrogen Corporation, Carlsbad, CA, USA) for the amplification of ribosomal protein L12 and PCR-*StyI* restriction fragment length polymorphism of the *omp2b* locus, as described (*11*,*12*).

**Figure 1:**
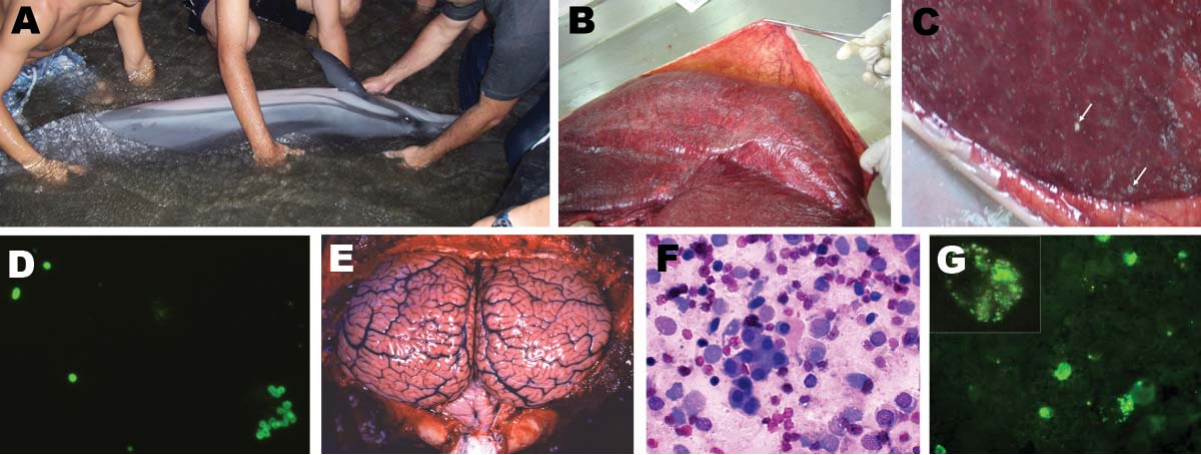
Clinical, pathologic, and immunofluorescence findings in stranded striped dolphin, *Stenella coeruleoalba.* A) Striped dolphin displaying swimming disorders being assisted by local persons; B) dolphin fetus within placenta; C) punctuated placental abscesses (arrows); D) immunofluorescent brucellae in impressions of placenta tissues; E) congested and hyperemic brain and cerebellum; F) Wright-Giemsa–stained mononuclear cell infiltrate in cerebrospinal fluid; G) immunofluorescent green *Brucella* spp. and *Brucella* debris within phagocytic cells infiltrating cerebrospinal fluid; the inset corresponds to an amplified phagocytic cell with fluorescent *Brucella* spp. and debris.

No penetrating wounds or mutilations were found in the dolphins. All animals displayed neurologic disorders characterized by the inability to maintain buoyancy and by opisthotonus, tremors, and seizures (Figure 1, panel **A**). In all animals, parasitosis was evident and included gastric, intestinal, and pulmonary nematodes as well as subblubber and retroperitoneal cestode larvae. Some dolphins had moderately to severely congested lungs, and 1 dolphin had splenomegaly. The pregnant female had milk in her mammary glands, a 66-cm fetus, and small placental abscesses (Figure 1, panels **B**, **C**). Hyperemic meninges, congested brain, and altered cerebrospinal fluid (augmented in volume and cellularity) were evident in all dolphins (Figure 1, panels **E**, **F**).

All dolphins had antibodies against *Brucella* LPS, as determined by immunofluorescence (>1:150 dilution), competitive ELISA (>60% positivity), and Western blot. With the exception of the female calf, all dolphins displayed positive Rose Bengal agglutination. In 6 dolphins, *Brucella* organisms were demonstrated by immunofluorescence of cerebrospinal fluids and thereafter in tissue impressions of the brain, spinal cord, lymph nodes, spleen, liver, and kidneys. Bacteria were isolated from the same organs and fluids. The pregnant animal had bacteria in the placenta (Figure 1, panel **D**), umbilical cord, milk, allantoic and amniotic fluids, and fetal tissues. A large number of phagocytes infiltrating the cerebrospinal fluid of these 6 dolphins contained intracellular *Brucella* organisms (Figure 1, panel **G**).

**Figure 2:**
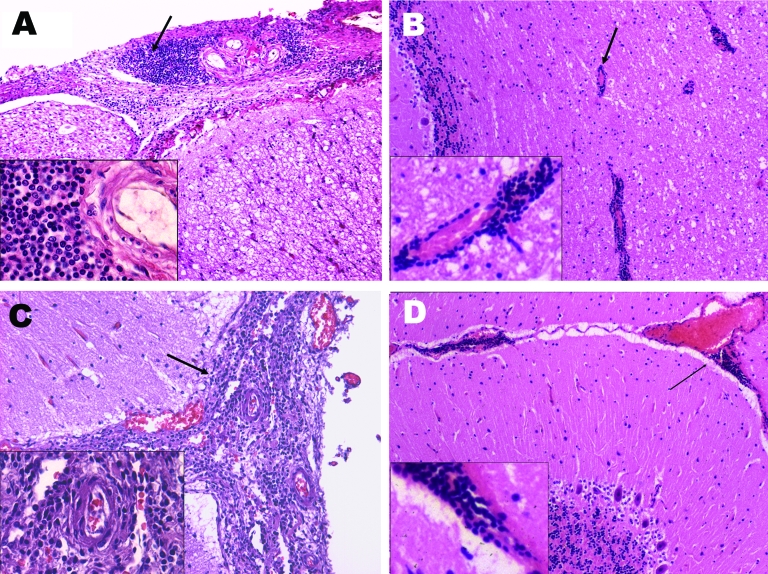
Main histopathologic finding of neurobrucellosis in *Stenella coeruleoalba.* A) Mononuclear infiltrates in the meninges (arrow) surrounding the spinal cord; B) mononuclear infiltrate around vessels (arrow) of the cerebellum; C) mononuclear infiltrate (arrow) in the meninges around the brain; D) hyperemic vessels and mononuclear cell infiltrate in the meninges around the cerebellum (arrow). The insets correspond to amplified sections of each figure demonstrating the mononuclear cell infiltrate.

Histopathologic examination of the central nervous system was performed on only the 6 dolphins from which *Brucella* organisms were isolated; tissues examined were spinal cord, medulla oblongata, cerebellum, pons, thalamic area, and the occipital and frontal cortices of the cerebrum. The most common and relevant histopathologic findings demonstrated meningoencephalitis (Figure 2) and were similar to those previously described (*13*). The predominant feature was nonsuppurative meningitis, which was more severe in the spinal cord, medulla oblongata, and cerebellum and somewhat more moderate and mild in the cerebral cortices. The meninges were hyperemic and, in most dolphins, edematous. Mild encephalitis was evidenced by a perivascular mononuclear infiltrate in the white and gray matter of the cerebrum, cerebellum, and brainstem and by a periventricular encephalitis that was widespread surrounding the third ventricle with the same cellular infiltrate found in the meninges. A major loss of ependyma was evident; a moderate to severe mononuclear choroiditis was also present. The predominant cellular infiltrate was composed of plasma cells, small lymphocytes, and macrophages. Little or no involvement of the neural tissue was noted in areas other than the periventricular zone. Vasculitis was not found.

Organisms compatible with *Brucella* (nonmotile; urease-, catalase-, and oxidase positive; gram-negative bacteria not requiring CO_2_ for growth) were isolated in blood agar or selective media from different organs, brain, and fluids from the 6 dolphins and the fetus subjected to detailed analysis. Standard bacteriologic, immunochemical, biochemical, and molecular typing indicated that the causative agent in all cases was *B. ceti* type I dolphin strain (Table).

**Table:** Characterization of *Brucella spp.* isolated from *Stenella coeruleoalba* and comparison with marine reference strains

Species, strain§					CO_2_¶	Serum against†			Growth on dyes, μg/mL‡								*Omp2b* restriction pattern
	RTD phage lysis*								Thionin					Basic fuchsin		O safranin	
	Tb	Wb	Iz	R/C		A	M		10	20	40	100		20		100	
*B. ceti,* B14/94#	–	+	+	–	–	+	+		+ (+)	+ (±)	+ (–)	+ (+)		+ (–)		+ (+)	Dolphin type I
*B. ceti*, B1/94#	–	+	–	–	–	+	–		+ (+)	+ (+)	+ (+)	+ (–)		+ (–)		+ (–)	Porpoise type II
*B. pinnipedialis,* B2/94#	–	–	+	–	+	+	–		+ (–)	+ (–)	+ (–)	+ (–)		+ (–)		+ (–)	Seal
*B. ceti*, B1 to B6**	–	+	+	–	–	+	+		+ (+)	+ (–)	– (–)	+ (–)		+ (–)		+ (–)	Dolphin type I

*RTD, routine test dilution of phages Tbilisi (Tb), Weybridge (Wb), Izatnagar (Iz), and rough type Wb derivative (R/C). †Serum against lipopolysaccharide epitopes, measured as agglutination with monospecific serum. ‡Dye concentrations expressed in µg/mL of culture medium with 10% CO_2_ or, within parenthesis, raw incubation without CO_2._ §All reference and isolated strains were SH_2_ negative, urease positive and amplified *Brucella spp* L12 rRNA by PCR. ¶Requirement. #Reference strains (*11*,*12*). **Isolates from Costa Rica.

## Conclusions

This report documents the presence of marine brucellosis along Latin American shorelines. The dolphins’ neurologic lesions were similar to those described previously in an infected *S. coeruleoalba* dolphin (*13*). This finding calls attention to possible increased susceptibility of this species to neurobrucellosis. Indeed, from a total of 46,826 individuals, corresponding to 28 cetacean species counted in the Costa Rican littorals, striped dolphins (inhabiting deep waters) represent only 13% of the animals sighted (*14*). From January 2004 through December 2006, the number of cetaceans reportedly stranded along the Pacific coast of Costa Rica was 31 (*15*). Of these, 14 were *S. coeruleoalba*; the other 16 comprised 11 species of odontocetes. During the 32-month period, we were able to collect samples: 10 (this study) from *S. coeruleoalba* and 1 from *K. sima*. This whale was negative for *Brucella* spp. infections. Endemic and migrating groups of *Delphinus delphis* and *Stenella attenuate* are found more frequently along the Costa Rican shorelines; however, few strandings of these species have been reported (*14*). The relatively high number of stranded *S. coeruleoalba* dolphins along the Costa Rican shorelines is in agreement with records of strandings along the European littorals (*11*). These results argue in favor of higher susceptibility of this species to neurobrucellosis.

The isolation of *B. ceti* from milk, fetal tissues, and secretions of a pregnant dolphin, and a similar discovery in European littorals, suggests that *B. ceti* is able to display tropism for placental and fetal tissues and that the bacteria may be shed, as it is with *Brucella*-infected livestock. This finding documents vertical transmission and the possibility of horizontal transmission to newborns. Moreover, the localization of the bacteria in particular organs suggests the possibility of transmission through sexual intercourse and may ensure the prevalence of both clinical and latent infections.

In terms of zoonotic transmission, we noticed that people handled and touched all these infected dolphins, mainly as an attempt to return them to the ocean. Other stranded dolphins have been transferred to privately owned swimming pools or to slaughter for use as a food source for humans and domestic animals. In this regard, susceptibility of *S. coeruleoalba* as reservoirs of *Brucella* spp. and modes of transmission must be taken into consideration.
