# Erratum—Vol. 14, No. 9

**DOI:** 10.3201/eid1410.071196b

**Published:** 2008-11

In Neurobrucellosis in Stranded Dolphins, Costa Rica (**G. Hernández-Mora et al.), the name of** co-author Elías Barquero-Calvo was misspelled. Several other editing changes to the online version of the article (available from www.cdc.gov/eid/content/14/9/1430.htm) have also been made upon the authors’ request.

